# Evaluation of the Worldwide Occurrence of Rabies in Dogs and Cats Using a Simple and Homogenous Framework for Quantitative Risk Assessments of Rabies Reintroduction in Disease-Free Areas through Pet Movements

**DOI:** 10.3390/vetsci7040207

**Published:** 2020-12-18

**Authors:** Guillaume Crozet, Julie Rivière, Laetitia Canini, Florence Cliquet, Emmanuelle Robardet, Barbara Dufour

**Affiliations:** 1 USC EPIMAI, ANSES, Ecole Nationale Vétérinaire d’Alfort, F-94700 Maisons-Alfort, France; julie.riviere@vet-alfort.fr (J.R.); barbara.dufour@vet-alfort.fr (B.D.); 2 Epidemiology Unit, Laboratory for Animal Health, ANSES, University Paris-Est, F-94700 Maisons-Alfort, France; laetitia.canini@anses.fr; 3 Nancy Laboratory for Rabies and Wildlife, ANSES, F-54220 Malzéville, France; florence.cliquet@anses.fr (F.C.); emmanuelle.robardet@anses.fr (E.R.)

**Keywords:** rabies, dog, cat, incidence, risk analysis, model, prediction

## Abstract

Dog and cat rabies cases imported from rabies enzootic countries represent a major threat for areas that have acquired rabies-free status and quantitative risk analyses (QRAs) are developed in order to assess this risk of rabies reintroduction through dog and cat movements. Herein we describe a framework to evaluate dog and cat rabies incidence levels in exporting countries along with the associated uncertainty for such QRAs. For enzootic dog rabies areas (EDRAs), we extended and adapted a previously published method to specify the relationship between dog rabies vaccination coverage and canine rabies incidence; the relationship between dog and cat rabies incidences; and then to predict annual dog and cat rabies incidences. In non-enzootic dog rabies areas (nEDRAs), we provided annual incidence based on declared dog and cat rabies cases. For EDRAs, we predicted an annual incidence potentially greater than 1.5% in dogs and about ten times lower in cats with a high burden in Africa and Asia but much lower in Latin America. In nEDRAs, the occurrence of rabies was lower and of similar magnitude in dogs and cats. However, wildlife could still potentially infect dogs and cats through spillover events. This framework can directly be incorporated in QRAs of rabies reintroduction.

## 1. Introduction

Rabies is a major and widespread zoonosis with a case-fatality rate of 100%, that causes approximately 60,000 human deaths each year [1]. In some areas such as Western Europe, Oceania or Japan the enzootic circulation of rabies (associated with Rabies Virus RABV) in domestic and wild animal populations has been halted, thus preventing human exposures. Nonetheless, rabies risk persists at low levels in these areas mostly because of (re)importations of rabies-infected animals, especially dogs and cats, from rabies enzootic areas [2]. In this context, it is crucial to assess the probability of rabies reintroduction through dog and cat movements in order to provide a deeper understanding of the processes responsible for the risk persistence. Moreover, models built to assess the probability of rabies reintroduction can be used to evaluate efficacy of risk mitigation measures or to test alternative scenarios of risk management. As a consequence, such risk assessments may be increasingly important in a context of globalisation, with more owners likely travelling with their pets [3,4,5]. An increasing number of countries with rabies-free status may also be interested in such analysis to define rabies risk management policies. Both quantitative and semi-quantitative risk assessment analyses have been implemented for rabies reintroduction in rabies-free areas [6,7,8,9,10,11,12,13,14,15,16,17].

In these quantitative risk assessments (QRAs), one crucial step has been and still is to provide rabies incidence in the animal population of interest (i.e., dogs and/or cats) in the exporting area(s). Incidence corresponds to the number of new cases of the disease of interest during a certain period of time (e.g., one year for annual incidence) divided by the number of individuals in the population [18]. The uncertainty is also key to depict this parameter accurately and can be specified by the variance of the parameter’s distribution [19]. The incidence will then define the risk level represented by one given animal species in one given area (or travelling to this area) [18], before considering implementation of risk mitigation measures (e.g., vaccination, serologic testing, border control). Providing accurate rabies incidence distributions can be challenging due to the scarcity, heterogeneity and variable reliability of available data to produce parameter distributions for QRA models. Various methodologies have been used to quantify dog and cat rabies incidences for the different QRAs of rabies reintroduction. Some rely on reported dog and cat rabies case counts by countries to official institutions (e.g., World Animal Health Organisation (OIE)) and rabies incidence is then modelled with a Gamma distribution to represent uncertainty of a mean number of events, occurring as a Poisson process, per unit time [8,11,12,13,14,16]. This method is convenient especially when dealing with worldwide dog and cat movements since these data are available for most of the countries. It is probably a reliable assumption for areas where rabies cases occur sporadically and where surveillance systems perform well as stated by the Great Britain Advisory Group on Quarantine [20]. Nonetheless, in some areas, mostly in enzootic dog rabies areas (EDRAs), there is an underreporting of animal rabies cases which highly depends on the considered country or region and its rabies surveillance system [21]. For example, an active surveillance programme in Kenya detected >70 times more rabid dogs than the existing passive surveillance system [22]. It has thus been proposed to use the maximum annual incidence over a several years period (e.g., 3 or 4 years) to maximise the risk [8,13]. However, the use of a maximum value in this context may not compensate for the level of underreporting. Others proposed to multiply these surveillance incidence data by a coefficient according to discrepancies observed between active and passive surveillance [15]. The use of such method to account for this underreporting bias is possible if animals included in the risk assessment of rabies reintroduction only come from a single country or area since this type of coefficient is adapted to a given surveillance system. Another drawback of using rabies case counts is the need to estimate dog and cat population sizes to obtain incidence. Dog and cat population sizes are difficult to estimate especially for areas with little information and can thus add more uncertainty in QRA models. Thus, despite being common in QRAs of rabies introduction, the use of declared rabies cases and animal population sizes could led to biased rabies incidences. It has also been proposed to use other data sources such as rabies incidences (i.e., number of new rabies cases divided by the population size of the investigated area) reported in scientific literature and as the result of specific investigations, often in a context of reinforced surveillance [10,20]. These data sources can then be used to produce non-parametric distributions (e.g., Uniform or Triangular distributions) directly from available rabies incidences [10] or by working from these reported incidence values completed with subject matter expert opinions [20]. It is noteworthy that such data sources avoid the need to use population size estimates.

In order to correctly evaluate rabies incidences, it is important to recall that rabies in dog and cat populations can have different epidemiological profiles, with a simple distinction that can be made between EDRAs and non-enzootic dog rabies areas (nEDRAs). In EDRAs, mainly in Africa and Asia, dogs are considered as the primary reservoir of rabies with high incidence levels. These countries have also the highest burden of human rabies, since dogs account for more than 95% of human rabies transmissions [1]. In these areas, research on the epidemiology of rabies in cats is less frequent, probably because of less significant public health implications since cats do not substantially contribute to human rabies exposures [1]. In EDRAs, cat rabies is nonetheless suspected to be closely related to dog rabies and the occurrence of cat rabies cases is assumed to be driven by the exposition to the dog reservoir [23,24,25]. In nEDRAs, such as North America, Europe, Oceania, some Asian Countries (e.g., Japan, Korea) or Southern Cone (e.g., Argentina, Chile, Uruguay and Paraguay), dogs do not act as the primary reservoir of rabies (as the result of control measures such as mass vaccination campaigns). However, sporadic cases of dog and cat rabies can still occur through spillover from wildlife (mainly from mesocarnivores or bats), if a wildlife rabies reservoir is present (e.g., North America, Eastern Europe, Southern Cone), or following the importation of dogs and cats from EDRAs as stated before [2,26,27,28,29,30,31].

Our objective was to develop a framework to provide annual dog and cat rabies incidences and their associated uncertainty in the different parts of the world. This framework also aimed at including the distinct rabies epidemiological profile of each area, classified as EDRAs and nEDRAs. This work was primarily designed to provide dog and cat rabies incidence distributions at the country or group of countries level for QRA models of rabies reintroduction in disease-free areas through pet movements. It could thus strengthen these models with the support of a framework taking into account the available state of knowledge about dog and cat rabies incidences worldwide and avoiding the use of poorly reliable sources of data when possible.

## 2. Materials and Methods

### 2.1. Framework Overview and Data Sources

**Figure 1:**
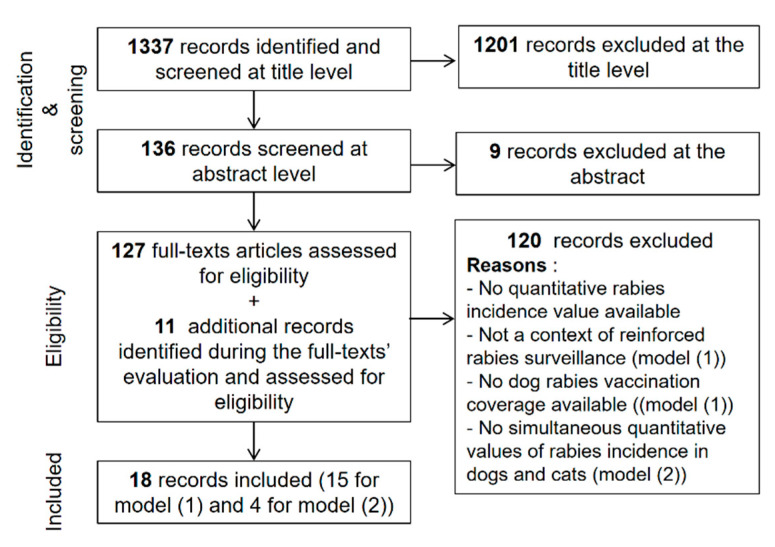
Literature search and records’ selection process to fit dog and cat rabies incidence prediction models in enzootic dog rabies areas. Model (1) and model (2) correspond to the annual dog rabies incidence and annual cat rabies incidence predictions models respectively.

To provide annual dog and cat rabies incidence values with their uncertainty for different areas of the world we distinguished EDRAs and nEDRAS given the two different underlying epidemiological contexts presented above. In EDRAs we developed prediction models based on a methodology initially proposed by Hampson et al. in 2015 [1] that was adapted and extended (especially for cat rabies incidence). To fit the two prediction models presented below, we conducted an extensive literature search to identify records providing dog and cat rabies incidence values. We searched the PubMed database using key words “rabies” in the title or the abstract AND “dog(s)” OR “canine” in the title or abstract AND “incidence” OR “prevalence”. Scientific articles published before November 2019 were investigated. The search results and the selection process are presented in Figure 1. 

We identified 18 publications (14 additional publications for the dog rabies incidence model [22,32,33,34,35,36,37,38,39,40,41,42,43,44,45,46,47,48,49,50], three publications for the cat rabies incidence model and one publication for both models [34,51,52,53,54,55]) that were used to complete the initial dataset provided by Hampson et al. in 2015 [1].

For nEDRAs, data-sources included declared dog and cat rabies case counts to official institutions (national or supra-national; e.g., OIE, PANAFTOSA-PAHO/WHO, Rabies-Bulletin-Europe) or publications reporting and compiling these data. Dog and cat population size estimates, necessary to compute incidence values when working with rabies case counts, were based on human:dog and human:cat ratios and were searched in the scientific and grey literature. Finally, human population sizes were extracted from the 2018 World Bank dataset [56].

### 2.2. EDRAs

- Model for dog rabies incidence prediction

In order to predict annual dog rabies incidence in different parts of the world, we used the functional relationship proposed by Hampson et al. in 2015 [1] which assumes that, in a given dog population, annual dog rabies incidence Idog depends on dog rabies vaccination coverage VC:

(1) $$Idog\;=\;{Idog}_{max}\times {\left(1-VC\right)}^{S1}$$

We estimated parameters $Idog_{max}$, the annual dog rabies incidence with non-existent vaccination coverage, and S1, a shape parameter, using annual dog rabies incidence time series available for Latin America and the associated dog rabies vaccination coverage previously used by Hampson et al. in 2015 [1] (48 observations). We supplemented it with data gathered from the 15 records identified in our literature search and reported annual dog rabies incidence and associated dog vaccination coverage in a context of reinforced surveillance in limited areas. Using these records, we added 30 observations to the initial dataset. This extended dataset reflected the various epidemiological contexts of rabies, most specifically with the data from studies carried out in Asia or Africa (see Table A1 for the extension of the initial dataset and for a list of references).

- Model for cat rabies incidence prediction

In EDRAs, we assumed that the annual incidence of cat rabies depends on the annual incidence of dog rabies, since dogs act as the primary reservoir [23,24,25]. Similarly to the annual dog rabies incidence model and according to the observed linear relationship between log-transformed raw data on annual dog and cat rabies incidences, we defined the functional relationship between cat and dog rabies incidences, Icat and Idog respectively, as follows:

(2) $$Icat=F\;\times \;Idog^{S2}$$

Parameters F, a multiplicative factor, and S2, a shape parameter, were estimated using reports that concurrently provided data on annual dog and cat rabies incidences for the same area identified in our literature search (detailed data and references are presented in Table A2). This model assumes equivalent rabies surveillance pressure on dogs and cats (i.e., equal probability of detection of rabies cases in the two species) since both cats and dogs are companion animals and no data in favour of different probabilities of detection were available.

The parameters of models (1) and (2) were estimated by maximum likelihood using the Broyden-Fletcher-Goldfarb-Shanno algorithm [57]. As previously stated by Hampson et al. [1], we assumed that residuals were Gamma-distributed. In other words, for each value of dog vaccination coverage in the model (1) and for each value of annual dog rabies incidence in the model (2), uncertainty was modelled by a Gamma distribution with specific shape and scale parameters: $\Gamma (k_{1},\frac{Idog}{k_{1}}\;)$ for model (1) and $\Gamma (k_{2},\;\frac{Icat}{k_{2}})$ for model (2); with $k_{1}$ and $k_{2}$, the shape parameters, being estimated along with other parameters by maximum likelihood.

- Prediction of dog and cat rabies incidences

**Figure 2:**
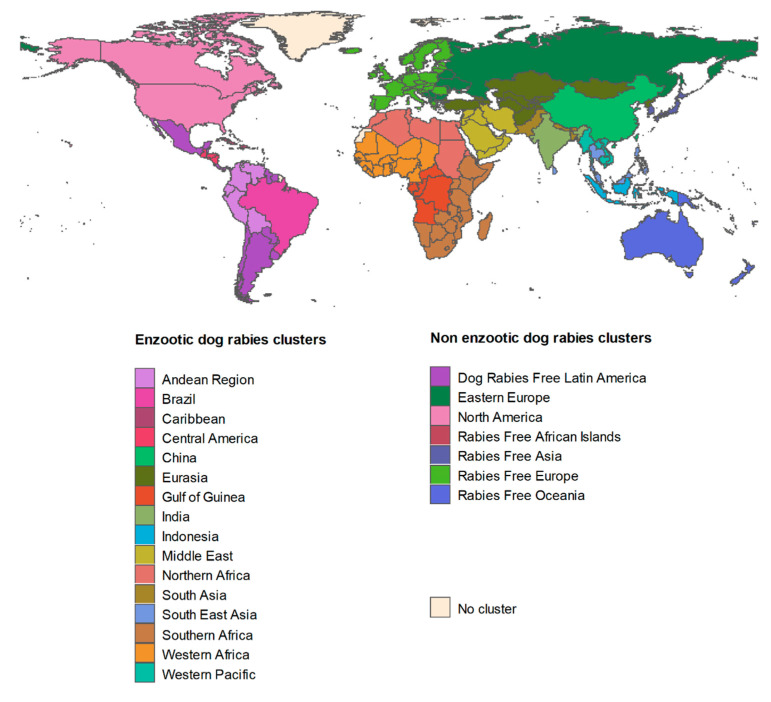
Clusters of countries used for the purpose of this study.

Annual dog rabies incidence was predicted using model (1) and dog vaccination coverage data from Hampson et al. in 2015 [1] provided for country or cluster of countries and obtained by expert elicitations. A “cluster” refers to countries grouped together due to their proximity, similar socioeconomic status and epidemiological rabies situation as previously described [1], adapted to factor in recent data and expert opinions. The 23 clusters (of which 16 are EDRAs) used here are presented in Figure 2. Annual cat rabies incidence was then predicted using dog rabies incidence predictions and model (2) both at country and cluster levels.

**Figure 3:**
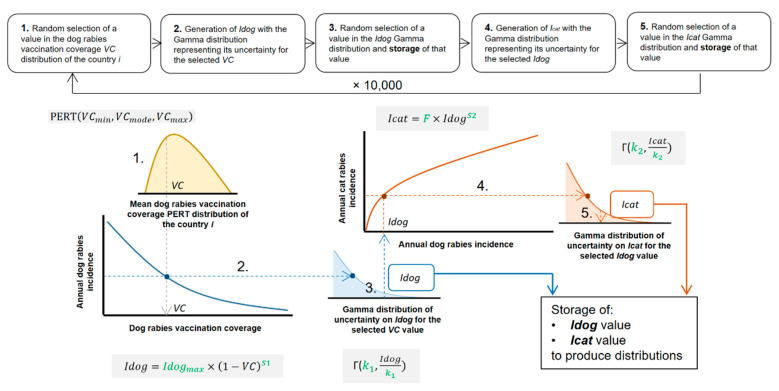
Iterative process to obtain the distributions for annual dog and cat rabies incidences in enzootic dog rabies areas. Parameters in green are estimated before running the iterative process (see the text for the definition of each parameter and the estimation method).

Uncertainty about the incidence predictions (i.e., distributions of Idog and Icat) was assessed for each cluster with the following iterative process which is also summarised in Figure 3: (1) Random selection of the dog rabies vaccination coverage using a PERT distribution (defined using minimum, modal and maximum values provided in [1]) for the chosen cluster; (2) Determination of the Gamma distribution parameters for dog rabies incidence using the selected level of vaccination coverage. The shape parameter $k_{1}$ was provided by the fitted model (1) and the scale parameter by the value of Idog (considered as the distribution mean) divided by the shape parameter; (3) Random selection and storage of a value in the generated annual dog rabies incidence distribution; (4) Determination of the Gamma distribution parameters for cat rabies incidence through the same process as in (2) using the selected level of annual dog rabies incidence but using model (2); (5) Random selection and storage of a value in the generated annual cat rabies incidence distribution. This iterative process was repeated 10,000 times.

### 2.3. nEDRAs

In nEDRAs (corresponding to seven out 23 clusters, see Figure 2) the assumption that dog rabies incidence depends on dog rabies vaccination coverage is not sustainable since the enzootic cycle is assumed to be disrupted and since rabies cases appear sporadically through exposure to another reservoir (i.e., wildlife) or through importations of infected pets from EDRAs. Similarly, cat rabies was supposed to be independent on dog rabies. In this specific context we used surveillance data, assuming no under-reporting. New dog and cat rabies cases were postulated to occur under a Poisson process and as previously used [8,11,13,16], we defined a Gamma distribution to model annual rabies incidence (a mean number of events per unit time, i.e., year) as follows [19]:

(3) $$\mathrm{Annual}\;\mathrm{rabies}\;\mathrm{incidence}\;\sim \;\frac{\Gamma (\mathrm{Number}\;\mathrm{of}\;\mathrm{rabies}\;\mathrm{cases}\;\mathrm{during}\;t\;,\;\frac{1}{t})}{\frac{\mathrm{Human}\;\mathrm{population}\;\mathrm{size}}{\mathrm{Human}:\mathrm{animal}\;\mathrm{ratio}}}$$

where t stands for the period (in years) during which rabies cases are enumerated. Uncertainty regarding human:animal ratios was taken into account. We used a PERT distribution if three or more ratios were available for the cluster using the mean of the ratios as modal value for this non-parametric distribution, the lowest value as minimum and the highest value as maximum. If only two ratios were available we defined a Uniform distribution using the values as extrema. Then 10,000 values were drawn from this distribution for each cluster of countries in nEDRAs.

Analyses were performed in the R environment (version R-3.6.1) [58] using R studio software [59]. Packages “bbmle” [57] for maximum likelihood estimates and “mc2d” [60] for PERT distributions and simulations were used.

## 3. Results

### 3.1. EDRAs

**Figure 4:**
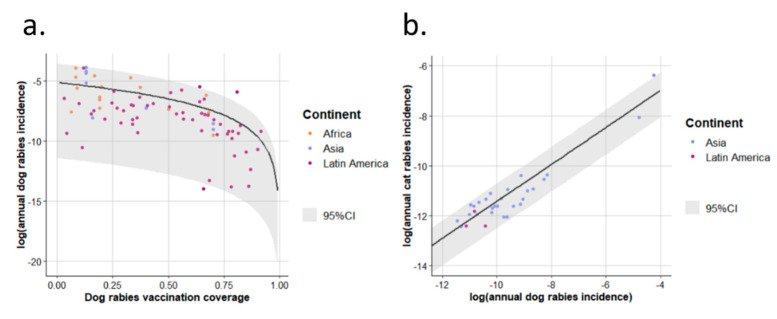
Model best fits for annual dog and cat rabies incidences in enzootic dog rabies areas. Model best fit for incidence of annual dog rabies is presented in (**a**) and that for annual cat rabies incidence in (**b**). The points represent the observations and are coloured by continents in which studies were conducted. Black lines represent best fits. 95%CI: 95% confidence interval of incidences (2.5th–97.5th percentile interval of the Gamma distribution of the residuals).

Reports concurrently providing annual dog rabies incidence and dog rabies vaccination coverage were used to fit model (1) and reports concurrently providing annual dog and cat rabies incidences were used to fit model (2) (see Section 2.2 and Appendix A).The parameter estimates were: $Idog_{max}$ = 5.73 × 10^−3^; S1 = 1.95; F = 1.78 × 10^−2^; S2 = 7.40 × 10^−1^ (Figure 4). The Gamma distribution shape parameters for the residuals of models (1) and (2) were respectively $k_{1}$ = 5.56 × 10^−1^ and $k_{2}$ = 5.30.

**Figure 5:**
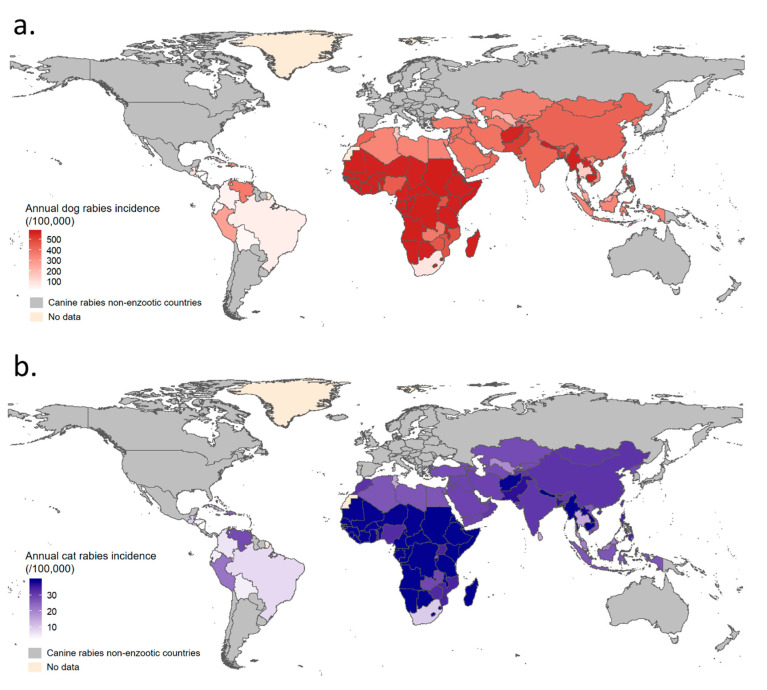
Mean predicted annual dog and cat rabies incidences by country in enzootic dog rabies areas. Map (**a**) for mean annual dog rabies incidence was produced using model (1) and map (**b**) for mean annual cat rabies incidence was produced using model (2). If dog rabies vaccination coverage was not available for a specific country, a global cluster estimate was used to predict canine rabies incidence.

Using these parameter estimates, mean annual dog and cat rabies incidences were then predicted for each country (Figure 5 and see Table A3 for detailed results). 

**Figure 6:**
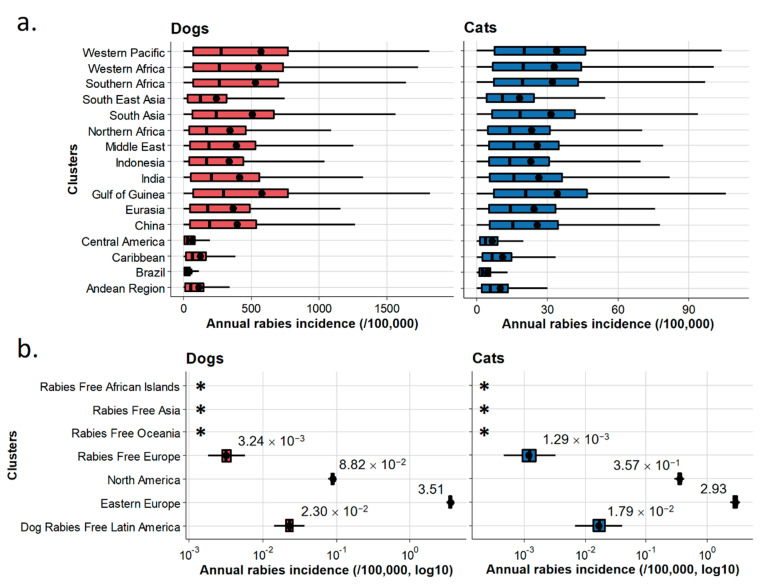
Annual dog and cat rabies incidences by cluster of countries. Annual incidence in enzootic dog rabies areas (**a**) and in non-enzootic dog rabies areas (**b**). Boxes represent the interquartile range (IQR); vertical bars represent the median; upper whiskers extend to the 75th percentile and the highest value that is within the 75th percentile + 1.5 × IQR; lower whiskers extend to the 25th percentile and the lowest value that is within the 25th percentile − 1.5 × IQR. Dots represent the mean annual incidence of the clusters (completed with mean values in (**b**)). *: no reported cases.

Annual dog and cat rabies incidences were then simulated at cluster level as shown in Figure 6a. The annual dog rabies incidence in EDRAs ranged from ~0 up to >1.5% (Figure 6a). The highest dog rabies incidences were predicted in Africa and Asia, where inter-country variability was greater due to heterogeneity in dog rabies vaccination coverage (e.g., mean predicted annual dog rabies incidence: 152/100,000 in Thailand versus 571/100,000 in Nepal). The predicted annual cat rabies incidence in EDRAs followed, in the proposed model, the same trends as annual canine rabies incidence but were tenfold lower than for dogs, ranging from ~0 up to ~1‰ in some African and Asian countries (Figure 5 and Figure 6a). As a result of high vaccination coverage, dog and cat rabies occurrence was predicted to be low in Latin America.

### 3.2. nEDRAs

Data gathered to compute incidences by using Equation (3) (i.e., rabies case counts and human:animal ratios) are summarised at the cluster level for the seven clusters considered as nEDRAs (Table 1). Declared dog and cat rabies case counts were collected from national or supranational organisms (OIE, PANAFTOSA-PAHO/WHO, Rabies-Bulletin-Europe, Centers for Disease Control and Prevention, Canadian Food Inspection Agency) for the 2013–2017 period. References used to define human:animal ratio distributions (PERT or Uniform) are also provided.

**Figure 7:**
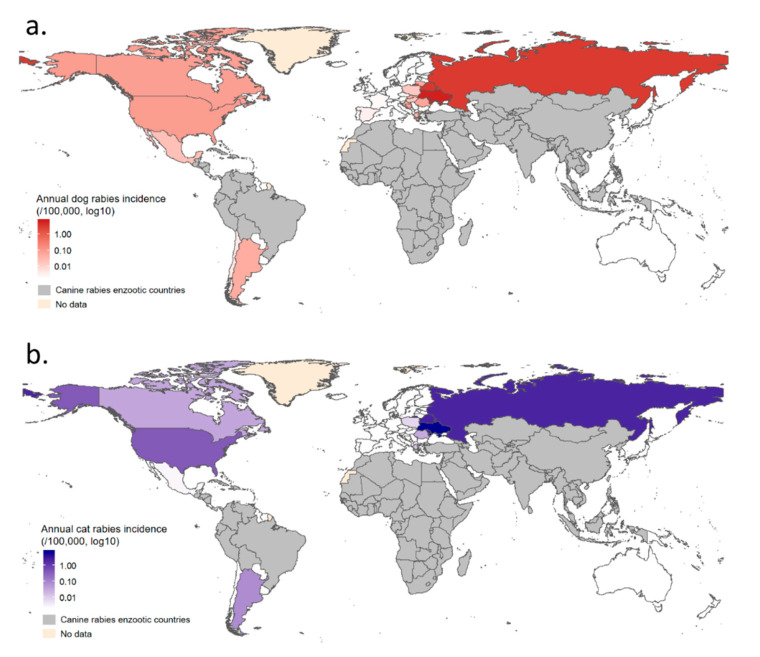
Mean annual dog and cat rabies incidences by country in non-enzootic dog rabies areas. Map (**a**) presents mean annual dog rabies and map (**b**) presents mean annual cat rabies incidence.

Results obtained for annual incidences in the seven clusters are reported in Figure 6b. Mean annual incidences are also presented by country using count data at the country level (Figure 7, see Table A4 for detailed results). Annual dog and cat rabies incidence values appeared to be low (<1/100,000 except for the Eastern Europe cluster where annual dog rabies incidence reached 3.51/100,000) even if wildlife can contribute to a number of spillover events in some clusters where a wildlife rabies reservoir exists (North America, Dog Rabies-Free Latin America, Eastern Europe). Rabies-Free Asia, Rabies Free African Islands and Rabies-Free Oceania clusters reported no imported cases contrary to Rabies-Free European cluster for the considered period, indicating heterogeneous risk levels associated with dog and cat translocations.

## 4. Discussion

We have proposed here a framework to define dogs and cats rabies incidence distributions as inputs for QRAs of rabies reintroduction in rabies-free areas through dog and cat movements. Provided parameters can directly be used to define QRA models. Moreover, such framework can allow updates if more data become available to fit the two prediction models for EDRAs, if other dog rabies vaccination coverage are applied or if other case counts (e.g., over other periods) are used to define Gamma distributions for nEDRAs. It is also important to highlight that the distinction between EDRAs and nEDRAs in this framework is a simplification of a more complex reality. For example in some nEDRAs rabies transmission chains between dogs could occur without persisting over time (e.g., in Eastern Europe where there are high levels of spillover from wildlife). Conversely, it is also possible that in EDRAs rabies transmission chains start to be disrupted because of high vaccination coverage, making the classification complex (e.g., In Latin America countries still reporting dog rabies, the incidence is low and transmission in dog populations is believed to be stopped soon [76]).

### 4.1. Model and Results for EDRAs

The method used for EDRAs is well suited for homogeneously evaluating annual dog and cat rabies incidences as it ignores discrepancies between countries in animal rabies surveillance and case reporting when using national surveillance reports [21]. The strength of our approach also lies in its ability to predict uncertainty (Figure 6), which is critical when performing quantitative risk analyses [19]. “Uncertainty” here is used in its broadest meaning since it accounts for both biological variability and limited knowledge and was particularly high in this context when incidence levels were high. However, bringing together studies carried out in different regions of the world illustrated various epidemiological contexts. Limited knowledge appeared to have an important impact when evaluating cat rabies occurrence for high dog rabies incidences since little information was available, leading to high-leverage data (Figure 4). It is also important to consider that this method, for the purpose of rabies reintroduction QRAs, aims to provide the global occurrence of rabies in dogs and cats at country or cluster level, but does not account for the spatial heterogeneity of rabies occurrence within a country or incidence time variations [77,78,79]. Spatial variability (e.g., as observed in Latin America with sometimes very focal areas where dog rabies is present [31]) and time variations are probably captured by the high level of uncertainty at low dog vaccination coverage for annual dog rabies incidence and, as a consequence, at high annual dog rabies incidences for annual cat rabies incidences. Also, animal subpopulation specificities (e.g., indoor pets versus free-roaming animals) could not be included in this model, whose purpose was to provide global incidence values at the country or cluster of countries level. Finally, in both models (1) and (2), the annual incidence of dog and cat rabies depends on only one variable, dog rabies vaccination coverage. To account for the effect of other covariates, more complex models need to be developed. Dog density and population turnover have been reported to be major predictors [77,79,80], but they could not be included in this framework since they were not available in most references. Such limitations were already identified by Hampson et al. [1]. Moreover, vaccination coverage in dog populations are sometimes difficult to estimate. To take this into account, we included uncertainty for this parameter when predicting incidences (by using a PERT distribution). However an overestimation of vaccination coverage would induce a lower rabies incidences in dogs and cats, and vice versa. Nonetheless, this method suits well the needs for quantitative risk analysis providing a homogenous way to provide annual rabies incidences for dogs and cats in exporting areas considered as EDRAs. Moreover, uncertainty associated with these values does not rely on non-parametric distributions arbitrarily chosen but aims at providing the actual level of knowledge and the biological variability observed in the field in an objective and standardised way. When compared to values used in risk analyses, we found similar results to QRAs also using similar data sources (i.e., incidence data obtained through investigations in a limited area with reinforced surveillance). For example, Hudson et al. in 2017 [10] used an Uniform distribution of 0.0001–0.05 (mean = 0.025) to model annual dog rabies incidence (i.e., annual probability of infection) in Indonesia which cover range of values similar to our results for Indonesia (Figure 6—mean: 3.38 × 10^−3^; 5th–95th percentiles: 2.07 × 10^−5^–1.27 × 10^−2^) but with a lower mean. However, another report using similar sources of data, produced non-parametric Triangular distributions (minimum, mode, maximum) with lower variance and with a lower range of values: for example they used a Triangular distribution (1 × 10^−5^; 1 × 10^−4^; 1 × 10^−3^) for Western Pacific while we obtained a mean dog rabies incidence of 5.66 × 10^−3^ (5th–95th percentiles: 4.09 × 10^−5^–2.06 × 10^−2^); or a Triangular distribution (1 × 10^−5^; 1 × 10^−4^; 1 × 10^−4^) for Sub Saharan Africa while we obtained a mean of 5.68 × 10^−3^ (5th–95th percentiles: 3.93 × 10^−5^–1.99 × 10^−2^) for Southern Africa. One explanation is that the authors decided to lower incidence level for the purpose of the QRAs assuming that imported dogs came from lower-risk areas within these EDRAs [20]. For QRAs using declared rabies counts and declared animal populations sizes in EDRAs, direct comparison is difficult. Indeed, data sources are of a different kind and incidence values are rarely directly provided since it is an intermediate step in the risk calculation. Nonetheless one QRA presented instantaneous prevalence (IP) (i.e., annual incidence multiplied by mean incubation period (≈35 days)/365 days) and the ranking based on these IPs between EDRAs seems different from our results (e.g., Caribbean had the second highest IP in this other study) [13]. The reason for such differences is not traceable since source of variation could arise from rabies case counts and/or dog and cat population size estimates.

### 4.2. Model and Results for nEDRAs

In nEDRAs, where cat rabies is not considered to be dependent on dog rabies, since cat rabies occurs through exposure to infected wildlife or the importation of infected pets (and not through exposure to infected dogs), the incidence in cats may be higher than the incidence in dogs. This implies that, in such contexts, the occurrence of dog and cat rabies is mainly related to the level of exposure to wildlife and that cats, especially in countries with a high Human Development Index (HDI) (e.g., North America), certainly roam freely more often than dogs [81]. According to this hypothesis, it was consistent to observe a similar annual dog and cat rabies incidence in countries with a lower HDI (e.g., Eastern Europe), where there are probably more free-roaming dogs [82]. For these incidence levels to be valid, we assumed that dog and cat rabies cases were well detected and reported in this context. This is a reasonable assumption since in nEDRAs, which often have a high HDI, dog and cat rabies occurs only sporadically because most dogs and cats are owned and surveillance systems are efficient. However, in case of animal rabies underreporting, we expect incidences to be underestimated with our framework. It is also worth noting that biased population sizes would affect the validity of incidence values. In order to limit the impact of this bias, we included uncertainty on human:animal ratios. The most common bias in the estimation of animal population sizes occurs when some categories of animals are ignored, e.g., non-owned dogs and cats, and leads to an underestimation of population sizes. Such underestimation would conduct to overestimated incidences. Nonetheless, this bias would have minor impact since incidence values were already very low in nEDRAs. Moreover, the use of this method to compute rabies incidences concerned a minority of clusters of countries (seven out of 23). When considering rabies-free areas with no wildlife rabies reservoir, very low residual rabies incidence (Rabies Free Europe) or absence of rabies occurrence (Rabies Free Asia, Rabies Free African Islands and Rabies Free Oceania) were observed. This discrepancy could arise from the implementation of different mitigation measures and the associated level of compliance or from different kinds of pet flows in terms of volume and origin to these rabies-free clusters. For rabies incidence in nEDRAs we found similar values and similar level of uncertainty to the other reports mentioning IPs (with respect to coefficient to obtain IP from incidence values) [13]. This is consistent with the fact that the same methodology was used in the specific case of nEDRAs, with a Gamma distribution to model a mean number of events per unit time (assuming new rabies cases follow a Poisson process); even if we used human:animal ratios to estimate dog and cat population sizes (allowing to follow human population growth) instead of raw counts. Non parametric distributions using similar data sources for nEDRAs produced same range of values but with more uncertainty (i.e., greater distribution variances). For example, the Great Britain Advisory Group on Quarantine [20] used for dog and cat rabies incidence in North America a Triangular distribution (0; 1 × 10^−6^; 1 × 10^−5^) which includes the range of values that we found when combining dog and cat rabies cases: mean incidence of 2.13 × 10^−6^ (5th–95th percentiles: 2.00 × 10^−6^–2.27 × 10^−6^).

## 5. Conclusions

The framework developed was used to evaluate annual dog and cat rabies incidences, along with their corresponding uncertainties, in the different parts of the world for the purpose of QRAs of rabies reintroduction in disease-free areas through dog and cat movements. In EDRAs (especially in African and Asian countries), we predicted an annual incidence of up to 1.5% in dogs and 1‰ in cats with high uncertainty levels. In nEDRAs, residual dog and cat rabies occurrence was observed due to infected dog and cat importations and, more importantly, to exposure to wildlife in areas where it is infected (with values >2/100,000 in Eastern Europe for example). In these areas incidence levels appeared to be similar in dogs and cats, or higher in cats compared to dogs. This framework can directly be implemented in QRAs of rabies reintroduction and avoid the use of non-parametric distributions to model dog and cat rabies incidences. We thus provide an objective approach to define parameters’ distributions for such models that take into account the current level of knowledge. More generally, such framework (and associated results) could also interest anyone wanting to obtain dog and cat rabies incidences at the country of or group of countries level.

## Figures and Tables

**Table 1:** Count data (2013–2017) and distributions used to compute dog and cat rabies incidences in non-enzootic dog rabies cluster of countries.

Cluster	Nature of the Residual Rabies Risk	Species	Number of Rabies Cases	Human:Animal Ratios	References
North America	Wildlife and importations	Dog	439	Uniform (4.26; 4.52)	[61,62] ^†^, [63,64] ^‡^
		Cat	1552	Uniform (4.46; 5.6)	
Rabies Free Asia	Importations	Dog	0	ND	[65] ^†^
		Cat	0	ND	
Rabies Free Oceania	Importations	Dog	0	ND	[65] ^†^
		Cat	0	ND	
Rabies Free Europe	Importations (and Wildlife)	Dog	23	PERT (4.86; 8.18; 16.2)	[66] ^†^, [67,68,69] ^‡^
		Cat	8	PERT (4.31; 7.47; 17.87)	
Eastern Europe	Wildlife and importations	Dog	5346	Uniform (8.26; 9.49)	[66] ^†^, [69] ^‡^
		Cat	5215	Uniform (6.42; 8.87)	
Dog Rabies Free Latin America	Wildlife and importations	Dog	63	PERT (3.40; 4.64; 6.40)	[70] ^†^, [71,72,73,74,75] ^‡^
		Cat	11	PERT (14.7; 20.56; 30.57) ^§^	
Rabies Free African Islands	Importations	Dog	0	ND	[65] ^†^
		Cat	0	ND	

ND: Not determined. For clusters with 0 rabies cases, animal population sizes were not evaluated since the numerator of Equation (3) will be 0. ^†^: Reference used to provide rabies case counts. ^‡^: Reference used to provide human:animal ratios. ^§^: For the “Dog Rabies Free Latin America” cluster, since few data were available, data from Brazil were also incorporated to define human:animal ratio distributions.

## Appendix A

**Table A1:** Data used to fit model (1) in addition to the initial dataset provided in Hampson et al. in 2015 [1].

City/Area	Country	Continent	Year	Annual Dog Rabies Incidence	Dog Rabies Vaccination Coverage	Reference
Gondar	Ethiopia	Africa	2009	4.12 × 10^−3^	0.2	[32]
Mongar	Bhutan	Asia	2005	1.23 × 10^−2^	0.13	[33] ^†^, [34]
Tashiyangtse	Bhutan	Asia	2005	1.44 × 10^−2^	0.13	[33] ^†^, [34]
Trashigang	Bhutan	Asia	2005	5.75 × 10^−3^	0.13	[33] ^†^, [34]
Serengeti	Tanzania	Africa	1997	3.71 × 10^−3^	0.091	[35]
Serengeti	Tanzania	Africa	1999	4.95 × 10^−4^	0.0639	[35]
Musoma	Tanzania	Africa	1997	1.89 × 10^−2^	0.085	[35]
Musoma	Tanzania	Africa	1999	9.02 × 10^−3^	0.085	[35]
Punjab	India	Asia	2016	3.05 × 10^−4^	0.16	[36]
N’Djamena	Chad	Africa	2001	1.40 × 10^−3^	0.19	[37]
Machakos	Kenya	Africa	1992	8.60 × 10^−3^	0.33	[22], [38] ^†^
Chukha	Bhutan	Asia	2008	2.00 × 10^−2^	0.13	[33] ^†^, [39]
Serengeti	Tanzania	Africa	2011	3.84 × 10^−3^	0.375	[40], [41] ^†^
Pemba	Tanzania	Africa	2011	1.03 × 10^−2^	0.168	[42]
Pemba	Tanzania	Africa	2016	4.88 × 10^−4^	0.682	[42]
N’Djamena	Chad	Africa	2012	7.00 × 10^−4^	0.19	[37] ^†^, [43]
N’Djamena	Chad	Africa	2014	7.30 × 10^−5^	0.7	[37] ^†^, [43]
Unguja	Tanzania	Africa	2018	6.15 × 10^−4^	0.5	[44]
N’Djamena	Chad	Africa	2016	2.00 × 10^−3^	0.67	[45]
N’Djamena	Chad	Africa	2006	1.71 × 10^−3^	0.19	[46], [47] ^†^
Bali Island	Bali	Asia	2008	6.75 × 10^−4^	0.4	[48] ^‡^, [49] (making the assumption of linear decrease for dog population between 2008 and 2019)
Bali Island	Bali	Asia	2010	1.94 × 10^−4^	0.7	[48] ^‡^, [49] (making the assumption of linear decrease for dog population between 2008 and 2019)
Bali Island	Bali	Asia	2011	1.16 × 10^−4^	0.7	[48] ^‡^, [49] (making the assumption of linear decrease for dog population between 2008 and 2019)
Hermosillo	Mexico	South Central America	1988	4.15 × 10^−3^	0.64	[50]
Hermosillo	Mexico	South Central America	1988	3.11 × 10^−3^	0.56	[50]
Hermosillo	Mexico	South Central America	1988	2.65 × 10^−3^	0.81	[50]
Hermosillo	Mexico	South Central America	1988	2.49 × 10^−3^	0.51	[50]
Hermosillo	Mexico	South Central America	1988	1.73 × 10^−3^	0.37	[50]
Hermosillo	Mexico	South Central America	1988	1.48 × 10^−3^	0.66	[50]
Hermosillo	Mexico	South Central America	1988	1.16 × 10^−3^	0.64	[50]

Annual incidence values correspond to an annual number of new rabies cases divided by a population size. ^†^: Reference specifically added to provide dog vaccination coverage. ^‡^: Reference specifically added to provide dog population size in order to calculate incidence.

**Table A2:** Data used to fit the model (2).

Area	Country	Continent	Year	Annual Cat Rabies Incidence	Annual Dog Rabies Incidence	Reference
Bangkok area	Thailand	Asia	1987	3.17 × 10^−5^	2.85 × 10^−4^	[51], [52] ^†^
Bangkok area	Thailand	Asia	1988	2.68 × 10^−5^	2.56 × 10^−4^	[51], [52] ^†^
Bangkok area	Thailand	Asia	1989	1.80 × 10^−5^	1.73 × 10^−4^	[51], [52] ^†^
Bangkok area	Thailand	Asia	1990	1.67 × 10^−5^	1.39 × 10^−4^	[51], [52] ^†^
Bangkok area	Thailand	Asia	1991	1.21 × 10^−5^	1.18 × 10^−4^	[51], [52] ^†^
Bangkok area	Thailand	Asia	1992	9.85 × 10^−6^	1.06 × 10^−4^	[51], [52] ^†^
Bangkok area	Thailand	Asia	1993	8.99 × 10^−6^	8.27 × 10^−5^	[51], [52] ^†^
Bangkok area	Thailand	Asia	1994	5.86 × 10^−6^	6.52 × 10^−5^	[51], [52] ^†^
Bangkok area	Thailand	Asia	1995	5.80 × 10^−6^	5.77 × 10^−5^	[51], [52] ^†^
Bangkok area	Thailand	Asia	1996	6.98 × 10^−6^	3.68 × 10^−5^	[51], [52] ^†^
Tashiyangtse	Bhutan	Asia	2005	1.73 × 10^−3^	1.44 × 10^−2^	[34]
Tashiyangtse	Bhutan	Asia	2006	3.20 × 10^−4^	8.42 × 10^−3^	[34]
State of Sao Paulo	Brazil	South America	1993	1.64 × 10^−6^	3.80 × 10^−6^	[53], [55] ^†^
State of Sao Paulo	Brazil	South America	1994	4.10 × 10^−6^	2.96 × 10^−5^	[53], [55] ^†^
State of Sao Paulo	Brazil	South America	1995	7.38 × 10^−6^	2.00 × 10^−5^	[53], [55] ^†^
State of Sao Paulo	Brazil	South America	1996	4.10 × 10^−6^	1.46 × 10^−5^	[53], [55] ^†^
Southern Thailand	Thailand	Asia	1994	3.08 × 10^−5^	1.08 × 10^−4^	[52,54]
Southern Thailand	Thailand	Asia	1995	1.74 × 10^−5^	6.62 × 10^−5^	[52] ^†^, [54]
Southern Thailand	Thailand	Asia	1996	1.24 × 10^−5^	5.24 × 10^−5^	[52] ^†^, [54]
Southern Thailand	Thailand	Asia	1997	8.94 × 10^−6^	4.23 × 10^−5^	[52] ^†^, [54]
Southern Thailand	Thailand	Asia	1998	8.44 × 10^−6^	3.77 × 10^−5^	[52] ^†^, [54]
Southern Thailand	Thailand	Asia	1999	8.94 × 10^−6^	4.57 × 10^−5^	[52] ^†^, [54]
Southern Thailand	Thailand	Asia	2000	1.49 × 10^−5^	3.58 × 10^−5^	[52] ^†^, [54]
Southern Thailand	Thailand	Asia	2001	1.19 × 10^−5^	3.01 × 10^−5^	[52] ^†^, [54]
Southern Thailand	Thailand	Asia	2002	1.04 × 10^−5^	2.32 × 10^−5^	[52] ^†^, [54]
Southern Thailand	Thailand	Asia	2003	6.46 × 10^−6^	2.19 × 10^−5^	[52] ^†^, [54]
Southern Thailand	Thailand	Asia	2004	9.44 × 10^−6^	1.73 × 10^−5^	[52] ^†^, [54]
Southern Thailand	Thailand	Asia	2005	8.94 × 10^−6^	1.94 × 10^−5^	[52] ^†^, [54]
Southern Thailand	Thailand	Asia	2006	6.46 × 10^−6^	1.66 × 10^−5^	[52] ^†^, [54]
Southern Thailand	Thailand	Asia	2007	3.97 × 10^−6^	1.20 × 10^−5^	[52] ^†^, [54]
Southern Thailand	Thailand	Asia	2008	4.97 × 10^−6^	1.05 × 10^−5^	[52] ^†^, [54]

Annual incidence values correspond to an annual number of new rabies cases divided by a population size. ^†^: Reference specifically added to provide animal population sizes in order to calculate incidence.

**Table A3:** Dog and cat annual incidences at the country level in EDRAs obtained using prediction models (1) and (2).

Country	Cluster	Dogs				Cats			
		Mean	Median	Interquartile Range		Mean	Median	Interquartile Range	
				25th Percentile	75th Percentile			25th Percentile	75th Percentile
**Algeria**	Northern Africa	3.38 × 10^−3^	1.66 × 10^−3^	4.25 × 10^−4^	4.50 × 10^−3^	2.65 × 10^−4^	1.36 × 10^−4^	4.80 × 10^−5^	9.37 × 10^−4^
**Djibouti**	Northern Africa	5.72 × 10^−3^	2.70 × 10^−3^	7.22 × 10^−4^	7.63 × 10^−3^	3.91 × 10^−4^	1.99 × 10^−4^	7.08 × 10^−5^	1.42 × 10^−3^
**Egypt**	Northern Africa	3.38 × 10^−3^	1.66 × 10^−3^	4.30 × 10^−4^	4.43 × 10^−3^	2.65 × 10^−4^	1.37 × 10^−4^	4.78 × 10^−5^	9.86 × 10^−4^
**Eritrea**	Northern Africa	5.72 × 10^−3^	2.81 × 10^−3^	7.38 × 10^−4^	7.60 × 10^−3^	3.91 × 10^−4^	2.02 × 10^−4^	7.26 × 10^−5^	1.41 × 10^−3^
**Libya**	Northern Africa	3.38 × 10^−3^	1.63 × 10^−3^	4.15 × 10^−4^	4.55 × 10^−3^	2.65 × 10^−4^	1.36 × 10^−4^	4.72 × 10^−5^	9.78 × 10^−4^
**Morocco**	Northern Africa	4.27 × 10^−3^	2.17 × 10^−3^	5.38 × 10^−4^	5.87 × 10^−3^	3.15 × 10^−4^	1.64 × 10^−4^	5.74 × 10^−5^	1.20 × 10^−3^
**Sudan**	Northern Africa	5.71 × 10^−3^	2.81 × 10^−3^	6.96 × 10^−4^	7.50 × 10^−3^	3.90 × 10^−4^	2.05 × 10^−4^	7.04 × 10^−5^	1.40 × 10^−3^
**Tunisia**	Northern Africa	1.58 × 10^−3^	7.76 × 10^−4^	2.06 × 10^−4^	2.13 × 10^−3^	1.51 × 10^−4^	7.82 × 10^−5^	2.83 × 10^−5^	5.33 × 10^−4^
**Angola**	Gulf of Guinea	5.68 × 10^−3^	2.85 × 10^−3^	7.17 × 10^−4^	7.83 × 10^−3^	3.89 × 10^−4^	2.08 × 10^−4^	7.20 × 10^−5^	1.45 × 10^−3^
**Burundi**	Gulf of Guinea	5.68 × 10^−3^	2.77 × 10^−3^	7.19 × 10^−4^	7.40 × 10^−3^	3.89 × 10^−4^	1.99 × 10^−4^	7.13 × 10^−5^	1.43 × 10^−3^
**Central African Republic**	Gulf of Guinea	5.68 × 10^−3^	2.88 × 10^−3^	7.50 × 10^−4^	7.83 × 10^−3^	3.89 × 10^−4^	2.08 × 10^−4^	7.42 × 10^−5^	1.49 × 10^−3^
**Congo**	Gulf of Guinea	5.72 × 10^−3^	2.75 × 10^−3^	6.90 × 10^−4^	7.74 × 10^−3^	3.91 × 10^−4^	2.03 × 10^−4^	6.97 × 10^−5^	1.42 × 10^−3^
**Congo (Dem. Rep.)**	Gulf of Guinea	5.68 × 10^−3^	2.87 × 10^−3^	7.08 × 10^−4^	7.66 × 10^−3^	3.89 × 10^−4^	2.07 × 10^−4^	7.01 × 10^−5^	1.45 × 10^−3^
**Equatorial Guinea**	Gulf of Guinea	5.68 × 10^−3^	2.90 × 10^−3^	7.45 × 10^−4^	7.75 × 10^−3^	3.89 × 10^−4^	2.09 × 10^−4^	7.44 × 10^−5^	1.48 × 10^−3^
**Gabon**	Gulf of Guinea	5.64 × 10^−3^	2.90 × 10^−3^	7.05 × 10^−4^	7.73 × 10^−3^	3.87 × 10^−4^	2.07 × 10^−4^	7.06 × 10^−5^	1.46 × 10^−3^
**Rwanda**	Gulf of Guinea	3.19 × 10^−3^	1.72 × 10^−3^	4.33 × 10^−4^	4.71 × 10^−3^	2.54 × 10^−4^	1.40 × 10^−4^	4.82 × 10^−5^	1.00 × 10^−3^
**Benin**	Western Africa	5.64 × 10^−3^	2.74 × 10^−3^	7.32 × 10^−4^	7.53 × 10^−3^	3.87 × 10^−4^	2.00 × 10^−4^	7.08 × 10^−5^	1.44 × 10^−3^
**Burkina**	Western Africa	5.64 × 10^−3^	2.60 × 10^−3^	6.67 × 10^−4^	7.19 × 10^−3^	3.87 × 10^−4^	1.93 × 10^−4^	6.74 × 10^−5^	1.38 × 10^−3^
**Cameroon**	Western Africa	5.64 × 10^−3^	2.82 × 10^−3^	6.95 × 10^−4^	7.68 × 10^−3^	3.87 × 10^−4^	2.08 × 10^−4^	7.01 × 10^−5^	1.40 × 10^−3^
**Chad**	Western Africa	5.64 × 10^−3^	2.61 × 10^−3^	6.67 × 10^−4^	7.16 × 10^−3^	3.87 × 10^−4^	1.91 × 10^−4^	6.90 × 10^−5^	1.38 × 10^−3^
**Cote d’Ivoire**	Western Africa	5.64 × 10^−3^	2.68 × 10^−3^	7.05 × 10^−4^	7.52 × 10^−3^	3.87 × 10^−4^	1.97 × 10^−4^	7.12 × 10^−5^	1.44 × 10^−3^
**Gambia**	Western Africa	5.64 × 10^−3^	2.79 × 10^−3^	7.02 × 10^−4^	7.46 × 10^−3^	3.87 × 10^−4^	2.03 × 10^−4^	7.09 × 10^−5^	1.38 × 10^−3^
**Ghana**	Western Africa	5.64 × 10^−3^	2.74 × 10^−3^	6.84 × 10^−4^	7.45 × 10^−3^	3.87 × 10^−4^	2.02 × 10^−4^	6.92 × 10^−5^	1.40 × 10^−3^
**Guinea**	Western Africa	5.64 × 10^−3^	2.70 × 10^−3^	6.92 × 10^−4^	7.24 × 10^−3^	3.87 × 10^−4^	1.98 × 10^−4^	6.90 × 10^−5^	1.35 × 10^−3^
**Guinea-Bissau**	Western Africa	5.64 × 10^−3^	2.67 × 10^−3^	7.21 × 10^−4^	7.13 × 10^−3^	3.87 × 10^−4^	1.96 × 10^−4^	7.15 × 10^−5^	1.36 × 10^−3^
**Liberia**	Western Africa	5.64 × 10^−3^	2.59 × 10^−3^	6.55 × 10^−4^	7.31 × 10^−3^	3.87 × 10^−4^	1.97 × 10^−4^	6.66 × 10^−5^	1.39 × 10^−3^
**Mali**	Western Africa	5.64 × 10^−3^	2.77 × 10^−3^	6.95 × 10^−4^	7.31 × 10^−3^	3.87 × 10^−4^	2.02 × 10^−4^	7.22 × 10^−5^	1.36 × 10^−3^
**Mauritania**	Western Africa	5.64 × 10^−3^	2.81 × 10^−3^	7.02 × 10^−4^	7.52 × 10^−3^	3.87 × 10^−4^	2.00 × 10^−4^	6.95 × 10^−5^	1.36 × 10^−3^
**Niger**	Western Africa	5.64 × 10^−3^	2.78 × 10^−3^	6.90 × 10^−4^	7.57 × 10^−3^	3.87 × 10^−4^	2.00 × 10^−4^	6.99 × 10^−5^	1.42 × 10^−3^
**Nigeria**	Western Africa	4.44 × 10^−3^	2.30 × 10^−3^	5.88 × 10^−4^	6.05 × 10^−3^	3.24 × 10^−4^	1.75 × 10^−4^	6.23 × 10^−5^	1.16 × 10^−3^
**Senegal**	Western Africa	5.64 × 10^−3^	2.79 × 10^−3^	6.89 × 10^−4^	7.51 × 10^−3^	3.87 × 10^−4^	2.01 × 10^−4^	7.07 × 10^−5^	1.39 × 10^−3^
**Sierra Leone**	Western Africa	5.64 × 10^−3^	2.74 × 10^−3^	7.25 × 10^−4^	7.25 × 10^−3^	3.87 × 10^−4^	2.00 × 10^−4^	7.06 × 10^−5^	1.37 × 10^−3^
**Togo**	Western Africa	4.85 × 10^−3^	2.43 × 10^−3^	6.07 × 10^−4^	6.42 × 10^−3^	3.46 × 10^−4^	1.84 × 10^−4^	6.25 × 10^−5^	1.25 × 10^−3^
**Botswana**	Southern Africa	5.67 × 10^−3^	2.76 × 10^−3^	6.65 × 10^−4^	7.22 × 10^−3^	3.89 × 10^−4^	2.01 × 10^−4^	6.88 × 10^−5^	1.38 × 10^−3^
**Ethiopia**	Southern Africa	5.67 × 10^−3^	2.70 × 10^−3^	6.90 × 10^−4^	7.08 × 10^−3^	3.89 × 10^−4^	1.94 × 10^−4^	6.85 × 10^−5^	1.42 × 10^−3^
**Kenya**	Southern Africa	5.67 × 10^−3^	2.64 × 10^−3^	7.20 × 10^−4^	7.05 × 10^−3^	3.89 × 10^−4^	1.96 × 10^−4^	7.06 × 10^−5^	1.37 × 10^−3^
**Lesotho**	Southern Africa	5.67 × 10^−3^	2.67 × 10^−3^	6.85 × 10^−4^	7.17 × 10^−3^	3.89 × 10^−4^	1.92 × 10^−4^	6.76 × 10^−5^	1.33 × 10^−3^
**Madagascar**	Southern Africa	5.67 × 10^−3^	2.66 × 10^−3^	6.54 × 10^−4^	7.15 × 10^−3^	3.89 × 10^−4^	1.97 × 10^−4^	6.77 × 10^−5^	1.35 × 10^−3^
**Malawi**	Southern Africa	5.67 × 10^−3^	2.66 × 10^−3^	6.59 × 10^−4^	7.27 × 10^−3^	3.89 × 10^−4^	2.00 × 10^−4^	6.75 × 10^−5^	1.34 × 10^−3^
**Mozambique**	Southern Africa	4.67 × 10^−3^	2.24 × 10^−3^	5.78 × 10^−4^	6.15 × 10^−3^	3.36 × 10^−4^	1.71 × 10^−4^	5.96 × 10^−5^	1.25 × 10^−3^
**Namibia**	Southern Africa	5.67 × 10^−3^	2.56 × 10^−3^	6.45 × 10^−4^	7.03 × 10^−3^	3.89 × 10^−4^	1.87 × 10^−4^	6.57 × 10^−5^	1.36 × 10^−3^
**Somalia**	Southern Africa	5.72 × 10^−3^	2.82 × 10^−3^	7.48 × 10^−4^	7.59 × 10^−3^	3.91 × 10^−4^	2.04 × 10^−4^	7.27 × 10^−5^	1.42 × 10^−3^
**South Africa**	Southern Africa	8.27 × 10^−4^	4.09 × 10^−4^	1.06 × 10^−4^	1.12 × 10^−3^	9.37 × 10^−5^	4.93 × 10^−5^	1.68 × 10^−5^	3.51 × 10^−4^
**Swaziland**	Southern Africa	5.67 × 10^−3^	2.70 × 10^−3^	6.50 × 10^−4^	7.09 × 10^−3^	3.89 × 10^−4^	1.96 × 10^−4^	6.76 × 10^−5^	1.35 × 10^−3^
**Uganda**	Southern Africa	4.67 × 10^−3^	2.31 × 10^−3^	6.05 × 10^−4^	6.23 × 10^−3^	3.36 × 10^−4^	1.77 × 10^−4^	6.28 × 10^−5^	1.22 × 10^−3^
**United Republic of Tanzania**	Southern Africa	5.67 × 10^−3^	2.69 × 10^−3^	6.79 × 10^−4^	7.11 × 10^−3^	3.89 × 10^−4^	1.98 × 10^−4^	6.94 × 10^−5^	1.33 × 10^−3^
**Zambia**	Southern Africa	3.71 × 10^−3^	2.00 × 10^−3^	5.15 × 10^−4^	5.42 × 10^−3^	2.84 × 10^−4^	1.59 × 10^−4^	5.63 × 10^−5^	1.08 × 10^−3^
**Zimbabwe**	Southern Africa	4.55 × 10^−3^	2.30 × 10^−3^	5.74 × 10^−4^	6.14 × 10^−3^	3.30 × 10^−4^	1.76 × 10^−4^	6.06 × 10^−5^	1.19 × 10^−3^
**Antigua and Barbuda**	Caribbean	1.20 × 10^−3^	6.04 × 10^−4^	1.53 × 10^−4^	1.61 × 10^−3^	1.24 × 10^−4^	6.59 × 10^−5^	2.29 × 10^−5^	4.45 × 10^−4^
**Cuba**	Caribbean	7.57 × 10^−4^	3.74 × 10^−4^	9.46 × 10^−5^	1.03 × 10^−3^	8.77 × 10^−5^	4.56 × 10^−5^	1.63 × 10^−5^	3.22 × 10^−4^
**Dominican Rep.**	Caribbean	3.13 × 10^−3^	1.56 × 10^−3^	3.96 × 10^−4^	4.23 × 10^−3^	2.50 × 10^−4^	1.33 × 10^−4^	4.56 × 10^−5^	8.98 × 10^−4^
**Haiti**	Caribbean	2.75 × 10^−3^	1.34 × 10^−3^	3.41 × 10^−4^	3.64 × 10^−3^	2.28 × 10^−4^	1.17 × 10^−4^	4.13 × 10^−5^	8.32 × 10^−4^
**El Salvador**	Central America	7.14 × 10^−4^	3.53 × 10^−4^	9.17 × 10^−5^	9.57 × 10^−4^	8.40 × 10^−5^	4.42 × 10^−5^	1.55 × 10^−5^	3.13 × 10^−4^
**Guatemala**	Central America	8.27 × 10^−4^	4.01 × 10^−4^	1.05 × 10^−4^	1.07 × 10^−3^	9.37 × 10^−5^	4.87 × 10^−5^	1.72 × 10^−5^	3.46 × 10^−4^
**Honduras**	Central America	1.93 × 10^−4^	9.00 × 10^−5^	2.24 × 10^−5^	2.53 × 10^−4^	3.20 × 10^−5^	1.60 × 10^−5^	5.49 × 10^−6^	1.20 × 10^−4^
**Nicaragua**	Central America	1.03 × 10^−4^	4.43 × 10^−5^	1.03 × 10^−5^	1.31 × 10^−4^	2.01 × 10^−5^	9.55 × 10^−6^	3.08 × 10^−6^	7.90 × 10^−5^
**Bolivia**	Andean Region	3.10 × 10^−4^	1.45 × 10^−4^	3.81 × 10^−5^	3.96 × 10^−4^	4.53 × 10^−5^	2.27 × 10^−5^	8.12 × 10^−6^	1.66 × 10^−4^
**Colombia**	Andean Region	4.41 × 10^−4^	2.18 × 10^−4^	5.62 × 10^−5^	6.28 × 10^−4^	5.89 × 10^−5^	3.11 × 10^−5^	1.09 × 10^−5^	2.31 × 10^−4^
**Ecuador**	Andean Region	2.88 × 10^−4^	1.77 × 10^−4^	4.48 × 10^−5^	5.01 × 10^−4^	4.30 × 10^−5^	2.61 × 10^−5^	9.18 × 10^−6^	1.93 × 10^−4^
**Peru**	Andean Region	2.65 × 10^−3^	1.16 × 10^−3^	2.95 × 10^−4^	3.24 × 10^−3^	2.21 × 10^−4^	1.05 × 10^−4^	3.70 × 10^−5^	7.78 × 10^−4^
**Venezuela**	Andean Region	3.66 × 10^−3^	1.40 × 10^−3^	3.57 × 10^−4^	3.89 × 10^−3^	2.81 × 10^−4^	1.20 × 10^−4^	4.28 × 10^−5^	9.15 × 10^−4^
**Brazil**	Brazil	5.92 × 10^−4^	2.90 × 10^−4^	7.35 × 10^−5^	7.96 × 10^−4^	7.31 × 10^−5^	3.76 × 10^−5^	1.31 × 10^−5^	2.71 × 10^−4^
**Cambodia**	Western Pacific	5.66 × 10^−3^	2.81 × 10^−3^	7.12 × 10^−4^	7.66 × 10^−3^	3.88 × 10^−4^	2.03 × 10^−4^	7.10 × 10^−5^	1.44 × 10^−3^
**Laos**	Western Pacific	5.58 × 10^−3^	2.77 × 10^−3^	6.78 × 10^−4^	7.40 × 10^−3^	3.84 × 10^−4^	2.03 × 10^−4^	6.98 × 10^−5^	1.43 × 10^−3^
**Myanmar**	Western Pacific	5.66 × 10^−3^	2.90 × 10^−3^	7.19 × 10^−4^	7.67 × 10^−3^	3.88 × 10^−4^	2.08 × 10^−4^	7.33 × 10^−5^	1.43 × 10^−3^
**Vietnam**	Western Pacific	3.27 × 10^−3^	1.50 × 10^−3^	3.89 × 10^−4^	4.21 × 10^−3^	2.59 × 10^−4^	1.29 × 10^−4^	4.49 × 10^−5^	9.45 × 10^−4^
**Bangladesh**	South Asia	5.18 × 10^−3^	2.45 × 10^−3^	6.39 × 10^−4^	6.65 × 10^−3^	3.63 × 10^−4^	1.87 × 10^−4^	6.61 × 10^−5^	1.30 × 10^−3^
**Bhutan**	South Asia	4.67 × 10^−3^	2.25 × 10^−3^	6.11 × 10^−4^	6.21 × 10^−3^	3.36 × 10^−4^	1.74 × 10^−4^	6.12 × 10^−5^	1.21 × 10^−3^
**Nepal**	South Asia	5.71 × 10^−3^	2.84 × 10^−3^	7.36 × 10^−4^	7.62 × 10^−3^	3.91 × 10^−4^	2.05 × 10^−4^	7.20 × 10^−5^	1.44 × 10^−3^
**Pakistan**	South Asia	5.18 × 10^−3^	2.45 × 10^−3^	5.85 × 10^−4^	6.86 × 10^−3^	3.63 × 10^−4^	1.81 × 10^−4^	6.16 × 10^−5^	1.30 × 10^−3^
**Malaysia**	South East Asia	2.38 × 10^−3^	1.16 × 10^−3^	3.06 × 10^−4^	3.24 × 10^−3^	2.05 × 10^−4^	1.06 × 10^−4^	3.65 × 10^−5^	7.62 × 10^−4^
**Philippines**	South East Asia	4.67 × 10^−3^	2.37 × 10^−3^	5.89 × 10^−4^	6.37 × 10^−3^	3.36 × 10^−4^	1.80 × 10^−4^	6.25 × 10^−5^	1.22 × 10^−3^
**Sri Lanka**	South East Asia	1.53 × 10^−3^	7.58 × 10^−4^	1.93 × 10^−4^	2.03 × 10^−3^	1.47 × 10^−4^	7.66 × 10^−5^	2.70 × 10^−5^	5.59 × 10^−4^
**Thailand**	South East Asia	1.52 × 10^−3^	7.47 × 10^−4^	1.87 × 10^−4^	2.04 × 10^−3^	1.47 × 10^−4^	7.73 × 10^−5^	2.65 × 10^−5^	5.49 × 10^−4^
**India**	India	4.18 × 10^−3^	2.02 × 10^−3^	4.89 × 10^−4^	5.52 × 10^−3^	3.10 × 10^−4^	1.58 × 10^−4^	5.41 × 10^−5^	1.12 × 10^−3^
**China**	China	4.25 × 10^−3^	1.86 × 10^−3^	4.76 × 10^−4^	5.10 × 10^−3^	3.14 × 10^−4^	1.51 × 10^−4^	5.30 × 10^−5^	1.06 × 10^−3^
**Indonesia**	Indonesia	3.38 × 10^−3^	1.65 × 10^−3^	4.20 × 10^−4^	4.52 × 10^−3^	2.65 × 10^−4^	1.36 × 10^−4^	4.72 × 10^−5^	9.80 × 10^−4^
**Bahrain**	Middle East	3.85 × 10^−3^	1.86 × 10^−3^	4.83 × 10^−4^	5.22 × 10^−3^	2.92 × 10^−4^	1.55 × 10^−4^	5.36 × 10^−5^	1.08 × 10^−3^
**Iran**	Middle East	3.88 × 10^−3^	1.94 × 10^−3^	4.87 × 10^−4^	5.33 × 10^−3^	2.93 × 10^−4^	1.55 × 10^−4^	5.23 × 10^−5^	1.03 × 10^−3^
**Iraq**	Middle East	3.85 × 10^−3^	1.86 × 10^−3^	4.70 × 10^−4^	5.07 × 10^−3^	2.92 × 10^−4^	1.49 × 10^−4^	5.19 × 10^−5^	1.03 × 10^−3^
**Israel**	Middle East	8.20 × 10^−4^	4.02 × 10^−4^	1.02 × 10^−4^	1.11 × 10^−3^	9.30 × 10^−5^	4.75 × 10^−5^	1.69 × 10^−5^	3.48 × 10^−4^
**Jordan**	Middle East	3.85 × 10^−3^	1.93 × 10^−3^	4.80 × 10^−4^	5.22 × 10^−3^	2.92 × 10^−4^	1.51 × 10^−4^	5.32 × 10^−5^	1.10 × 10^−3^
**Kuwait**	Middle East	3.85 × 10^−3^	1.94 × 10^−3^	4.79 × 10^−4^	5.13 × 10^−3^	2.92 × 10^−4^	1.51 × 10^−4^	5.31 × 10^−5^	1.04 × 10^−3^
**Lebanon**	Middle East	3.85 × 10^−3^	1.85 × 10^−3^	4.65 × 10^−4^	5.20 × 10^−3^	2.92 × 10^−4^	1.50 × 10^−4^	5.24 × 10^−5^	1.07 × 10^−3^
**Oman**	Middle East	4.18 × 10^−3^	2.12 × 10^−3^	5.25 × 10^−4^	5.61 × 10^−3^	3.10 × 10^−4^	1.61 × 10^−4^	5.64 × 10^−5^	1.13 × 10^−3^
**Qatar**	Middle East	3.85 × 10^−3^	1.94 × 10^−3^	4.78 × 10^−4^	5.25 × 10^−3^	2.92 × 10^−4^	1.56 × 10^−4^	5.35 × 10^−5^	1.06 × 10^−3^
**Saudi Arabia**	Middle East	3.85 × 10^−3^	1.86 × 10^−3^	4.81 × 10^−4^	5.04 × 10^−3^	2.92 × 10^−4^	1.50 × 10^−4^	5.34 × 10^−5^	1.10 × 10^−3^
**Syria**	Middle East	3.85 × 10^−3^	1.87 × 10^−3^	4.77 × 10^−4^	5.33 × 10^−3^	2.92 × 10^−4^	1.51 × 10^−4^	5.26 × 10^−5^	1.08 × 10^−3^
**United Arab Emirates**	Middle East	3.85 × 10^−3^	1.93 × 10^−3^	5.12 × 10^−4^	5.16 × 10^−3^	2.92 × 10^−4^	1.54 × 10^−4^	5.50 × 10^−5^	1.09 × 10^−3^
**Yemen**	Middle East	3.85 × 10^−3^	1.94 × 10^−3^	4.73 × 10^−4^	5.16 × 10^−3^	2.92 × 10^−4^	1.55 × 10^−4^	5.21 × 10^−5^	1.08 × 10^−3^
**Turkey**	Eurasia	3.62 × 10^−3^	1.78 × 10^−3^	4.38 × 10^−4^	4.69 × 10^−3^	2.79 × 10^−4^	1.44 × 10^−4^	4.94 × 10^−5^	1.02 × 10^−3^
**Armenia**	Eurasia	3.62 × 10^−3^	1.84 × 10^−3^	4.44 × 10^−4^	4.87 × 10^−3^	2.79 × 10^−4^	1.50 × 10^−4^	4.98 × 10^−5^	1.03 × 10^−3^
**Azerbaijan**	Eurasia	3.62 × 10^−3^	1.71 × 10^−3^	4.33 × 10^−4^	4.83 × 10^−3^	2.79 × 10^−4^	1.41 × 10^−4^	4.84 × 10^−5^	1.02 × 10^−3^
**Georgia**	Eurasia	3.62 × 10^−3^	1.76 × 10^−3^	4.60 × 10^−4^	4.87 × 10^−3^	2.79 × 10^−4^	1.45 × 10^−4^	5.05 × 10^−5^	1.03 × 10^−3^
**Afghanistan**	Eurasia	5.62 × 10^−3^	2.76 × 10^−3^	7.40 × 10^−4^	7.50 × 10^−3^	3.86 × 10^−4^	2.04 × 10^−4^	7.34 × 10^−5^	1.43 × 10^−3^
**Korea (north)**	Eurasia	3.62 × 10^−3^	1.85 × 10^−3^	4.52 × 10^−4^	4.88 × 10^−3^	2.79 × 10^−4^	1.48 × 10^−4^	5.13 × 10^−5^	1.02 × 10^−3^
**Kazakhstan**	Eurasia	3.62 × 10^−3^	1.75 × 10^−3^	4.47 × 10^−4^	4.76 × 10^−3^	2.79 × 10^−4^	1.44 × 10^−4^	5.15 × 10^−5^	1.02 × 10^−3^
**Kyrgyzstan**	Eurasia	3.62 × 10^−3^	1.77 × 10^−3^	4.50 × 10^−4^	4.83 × 10^−3^	2.79 × 10^−4^	1.44 × 10^−4^	5.02 × 10^−5^	1.04 × 10^−3^
**Mongolia**	Eurasia	4.18 × 10^−3^	2.14 × 10^−3^	5.60 × 10^−4^	5.74 × 10^−3^	3.10 × 10^−4^	1.70 × 10^−4^	5.84 × 10^−5^	1.14 × 10^−3^
**Tajikistan**	Eurasia	4.20 × 10^−3^	2.10 × 10^−3^	5.43 × 10^−4^	5.70 × 10^−3^	3.11 × 10^−4^	1.65 × 10^−4^	5.78 × 10^−5^	1.17 × 10^−3^
**Turkmenistan**	Eurasia	3.62 × 10^−3^	1.76 × 10^−3^	4.57 × 10^−4^	4.90 × 10^−3^	2.79 × 10^−4^	1.46 × 10^−4^	5.10 × 10^−5^	1.02 × 10^−3^
**Uzbekistan**	Eurasia	2.10 × 10^−3^	1.05 × 10^−3^	2.58 × 10^−4^	2.87 × 10^−3^	1.86 × 10^−4^	9.93 × 10^−5^	3.38 × 10^−5^	7.26 × 10^−4^

Annual incidence values correspond to an annual number of new rabies cases divided by a population size.

**Table A4:** Dog and cat annual incidences at the country level in nEDRAs obtained using Equation (3).

Country	Cluster	Dogs				Cats			
		Mean	Median	Interquartile Range		Mean	Median	Interquartile Range	
				25th Percentile	75th Percentile			25th Percentile	75th Percentile
Argentina	Dog Rabies Free Latin America	4.78 × 10^−7^	4.70 × 10^−7^	4.00 × 10^−7^	5.46 × 10^−7^	7.92 × 10^−7^	7.58 × 10^−7^	5.90 × 10^−7^	1.42 × 10^−6^
Chile	Dog Rabies Free Latin America	4.20 × 10^−8^	2.89 × 10^−8^	1.21 × 10^−8^	5.79 × 10^−8^	0	0	0	0
Paraguay	Dog Rabies Free Latin America	0	0	0	0	0	0	0	0
Uruguay	Dog Rabies Free Latin America	0	0	0	0	0	0	0	0
Costa Rica	Dog Rabies Free Latin America	0	0	0	0	0	0	0	0
Panama	Dog Rabies Free Latin America	0	0	0	0	0	0	0	0
Bahamas	Dog Rabies Free Latin America	0	0	0	0	0	0	0	0
Barbados	Dog Rabies Free Latin America	0	0	0	0	0	0	0	0
Dominica	Dog Rabies Free Latin America	0	0	0	0	0	0	0	0
Grenada	Dog Rabies Free Latin America	0	0	0	0	0	0	0	0
Jamaica	Dog Rabies Free Latin America	0	0	0	0	0	0	0	0
St. Kitts & Nevis	Dog Rabies Free Latin America	0	0	0	0	0	0	0	0
St. Lucia	Dog Rabies Free Latin America	0	0	0	0	0	0	0	0
St. Vincent & the Grenadines	Dog Rabies Free Latin America	0	0	0	0	0	0	0	0
Trinidad & Tobago	Dog Rabies Free Latin America	0	0	0	0	0	0	0	0
Belize	Dog Rabies Free Latin America	0	0	0	0	0	0	0	0
Guyana	Dog Rabies Free Latin America	0	0	0	0	0	0	0	0
Suriname	Dog Rabies Free Latin America	0	0	0	0	0	0	0	0
Mexico	Dog Rabies Free Latin America	2.19 × 10^−7^	2.15 × 10^−7^	1.87 × 10^−7^	2.47 × 10^−7^	2.86 × 10^−8^	1.95 × 10^−8^	7.92 × 10^−9^	1.08 × 10^−7^
Canada	North America	8.12 × 10^−7^	8.03 × 10^−7^	7.21 × 10^−7^	8.93 × 10^−7^	3.39 × 10^−7^	3.31 × 10^−7^	2.74 × 10^−7^	5.41 × 10^−7^
United States	North America	8.89 × 10^−7^	8.88 × 10^−7^	8.57 × 10^−7^	9.20 × 10^−7^	3.94 × 10^−6^	3.93 × 10^−6^	3.71 × 10^−6^	4.44 × 10^−6^
Japan	Rabies Free Asia	0	0	0	0	0	0	0	0
Maldives	Rabies Free Asia	0	0	0	0	0	0	0	0
Korea (south)	Rabies Free Asia	0	0	0	0	0	0	0	0
Singapore	Rabies Free Asia	0	0	0	0	0	0	0	0
Brunei	Rabies Free Asia	0	0	0	0	0	0	0	0
East Timor	Rabies Free Asia	0	0	0	0	0	0	0	0
Australia	Rabies Free Oceania	0	0	0	0	0	0	0	0
Cook islands	Rabies Free Oceania	0	0	0	0	0	0	0	0
Fiji	Rabies Free Oceania	0	0	0	0	0	0	0	0
Kiribati	Rabies Free Oceania	0	0	0	0	0	0	0	0
Marshall Islands	Rabies Free Oceania	0	0	0	0	0	0	0	0
Micronesia	Rabies Free Oceania	0	0	0	0	0	0	0	0
Nauru	Rabies Free Oceania	0	0	0	0	0	0	0	0
New Zealand	Rabies Free Oceania	0	0	0	0	0	0	0	0
Niue	Rabies Free Oceania	0	0	0	0	0	0	0	0
Palau	Rabies Free Oceania	0	0	0	0	0	0	0	0
Papua New Guinea	Rabies Free Oceania	0	0	0	0	0	0	0	0
Samoa	Rabies Free Oceania	0	0	0	0	0	0	0	0
Solomon Islands	Rabies Free Oceania	0	0	0	0	0	0	0	0
Tonga	Rabies Free Oceania	0	0	0	0	0	0	0	0
Tuvalu	Rabies Free Oceania	0	0	0	0	0	0	0	0
Vanuatu	Rabies Free Oceania	0	0	0	0	0	0	0	0
Andorra	Rabies Free Europe	0	0	0	0	0	0	0	0
Austria	Rabies Free Europe	0	0	0	0	0	0	0	0
Belgium	Rabies Free Europe	0	0	0	0	0	0	0	0
Croatia	Rabies Free Europe	3.67 × 10^−7^	2.43 × 10^−7^	1.01 × 10^−7^	5.03 × 10^−7^	0	0	0	0
Cyprus	Rabies Free Europe	0	0	0	0	0	0	0	0
Czech Republic	Rabies Free Europe	0	0	0	0	0	0	0	0
Denmark	Rabies Free Europe	0	0	0	0	0	0	0	0
Estonia	Rabies Free Europe	0	0	0	0	0	0	0	0
Finland	Rabies Free Europe	0	0	0	0	0	0	0	0
France	Rabies Free Europe	2.23 × 10^−8^	1.50 × 10^−8^	6.23 × 10^−9^	3.01 × 10^−8^	2.19 × 10^−8^	1.43 × 10^−8^	5.94 × 10^−9^	8.68 × 10^−8^
Germany	Rabies Free Europe	1.76 × 10^−8^	1.18 × 10^−8^	4.79 × 10^−9^	2.37 × 10^−8^	0	0	0	0
Greece	Rabies Free Europe	4.17 × 10^−7^	3.62 × 10^−7^	2.29 × 10^−7^	5.45 × 10^−7^	1.36 × 10^−7^	8.97 × 10^−8^	3.83 × 10^−8^	5.45 × 10^−7^
Hungary	Rabies Free Europe	1.52 × 10^−7^	1.01 × 10^−7^	4.15 × 10^−8^	2.08 × 10^−7^	0	0	0	0
Iceland	Rabies Free Europe	0	0	0	0	0	0	0	0
Ireland	Rabies Free Europe	0	0	0	0	0	0	0	0
Italy	Rabies Free Europe	0	0	0	0	0	0	0	0
Latvia	Rabies Free Europe	0	0	0	0	0	0	0	0
Lithuania	Rabies Free Europe	5.36 × 10^−7^	3.52 × 10^−7^	1.46 × 10^−7^	7.25 × 10^−7^	0	0	0	0
Luxembourg	Rabies Free Europe	0	0	0	0	0	0	0	0
Malta	Rabies Free Europe	0	0	0	0	0	0	0	0
Monaco	Rabies Free Europe	0	0	0	0	0	0	0	0
Netherlands	Rabies Free Europe	0	0	0	0	0	0	0	0
Norway	Rabies Free Europe	0	0	0	0	0	0	0	0
Poland	Rabies Free Europe	1.59 × 10^−7^	1.30 × 10^−7^	7.16 × 10^−8^	2.12 × 10^−7^	7.77 × 10^−8^	5.09 × 10^−8^	2.06 × 10^−8^	3.04 × 10^−7^
Portugal	Rabies Free Europe	0	0	0	0	0	0	0	0
Romania	Rabies Free Europe	6.14 × 10^−7^	5.39 × 10^−7^	3.68 × 10^−7^	7.85 × 10^−7^	3.01 × 10^−7^	2.40 × 10^−7^	1.33 × 10^−7^	9.27 × 10^−7^
San Marino	Rabies Free Europe	0	0	0	0	0	0	0	0
Slovakia	Rabies Free Europe	5.46 × 10^−7^	4.43 × 10^−7^	2.49 × 10^−7^	7.29 × 10^−7^	0	0	0	0
Slovenia	Rabies Free Europe	0	0	0	0	0	0	0	0
Spain	Rabies Free Europe	3.19 × 10^−8^	2.16 × 10^−8^	8.82 × 10^−9^	4.36 × 10^−8^	0	0	0	0
Sweden	Rabies Free Europe	0	0	0	0	0	0	0	0
Switzerland	Rabies Free Europe	0	0	0	0	0	0	0	0
United Kingdom	Rabies Free Europe	0	0	0	0	0	0	0	0
Albania	Eastern Europe	0	0	0	0	0	0	0	0
Belarus	Eastern Europe	3.50 × 10^−5^	3.48 × 10^−5^	3.30 × 10^−5^	3.68 × 10^−5^	2.33 × 10^−5^	2.31 × 10^−5^	2.12 × 10^−5^	2.88 × 10^−5^
Bosnia and Herzegovina	Eastern Europe	8.98 × 10^−7^	7.50 × 10^−7^	4.38 × 10^−7^	1.20 × 10^−6^	0	0	0	0
Bulgaria	Eastern Europe	0	0	0	0	0	0	0	0
Moldova	Eastern Europe	6.69 × 10^−5^	6.67 × 10^−5^	6.26 × 10^−5^	7.08 × 10^−5^	2.83 × 10^−5^	2.80 × 10^−5^	2.53 × 10^−5^	3.66 × 10^−5^
Serbia	Eastern Europe	0	0	0	0	0	0	0	0
Macedonia	Eastern Europe	0	0	0	0	0	0	0	0
Ukraine	Eastern Europe	6.34 × 10^−5^	6.34 × 10^−5^	6.11 × 10^−5^	6.56 × 10^−5^	7.14 × 10^−5^	7.14 × 10^−5^	6.59 × 10^−5^	8.25 × 10^−5^
Russian Federation	Eastern Europe	3.12 × 10^−5^	3.12 × 10^−5^	3.01 × 10^−5^	3.22 × 10^−5^	2.15 × 10^−5^	2.15 × 10^−5^	1.98 × 10^−5^	2.49 × 10^−5^
Montenegro	Eastern Europe	0	0	0	0	0	0	0	0
Cape Verde	Rabies Free African Islands	0	0	0	0	0	0	0	0
Sao Tome and Principe	Rabies Free African Islands	0	0	0	0	0	0	0	0
Comoros	Rabies Free African Islands	0	0	0	0	0	0	0	0
Mauritius	Rabies Free African Islands	0	0	0	0	0	0	0	0
Seychelles	Rabies Free African Islands	0	0	0	0	0	0	0	0

Annual incidence values correspond to an annual number of new rabies cases divided by a population size.
